# CdsH Contributes to the Replication of *Salmonella* Typhimurium inside Epithelial Cells in a Cysteine-Supplemented Medium

**DOI:** 10.3390/microorganisms8122019

**Published:** 2020-12-17

**Authors:** Fernando Díaz-Yáñez, Ricardo Álvarez, Iván L. Calderón, Juan A. Fuentes, Fernando Gil

**Affiliations:** 1 Microbiota-Host Interactions and Clostridia Research Group, Departamento de Ciencias Biológicas, Facultad de Ciencias de la Vida, Universidad Andres Bello, 8370186 Santiago, Chile; f.diazyanez@outlook.com (F.D.-Y.); rh.alvareze@gmail.com (R.Á.); 2 ANID-Millennium Science Initiative Program-Millennium Nucleus in the Biology of the Intestinal Microbiota, 8370186 Santiago, Chile; 3 Laboratorio de RNAs Bacterianos, Departamento de Ciencias Biológicas, Facultad de Ciencias de la Vida, Universidad Andres Bello, 8370186 Santiago, Chile; lcalderon@unab.cl; 4 Laboratorio de Genética y Patogénesis Bacteriana, Departamento de Ciencias Biológicas, Facultad de Ciencias de la Vida, Universidad Andres Bello, 8370186 Santiago, Chile

**Keywords:** *cdsH* expression, cysteine toxicity, epithelial cell infection

## Abstract

*Salmonella* Typhimurium is a facultative, intracellular pathogen whose products range from self-limited gastroenteritis to systemic diseases. Food ingestion increases biomolecules’ concentration in the intestinal lumen, including amino acids such as cysteine, which is toxic in a concentration-dependent manner. When cysteine’s intracellular concentration reaches toxic levels, *S*. Typhimurium expresses a cysteine-inducible enzyme (CdsH), which converts cysteine into pyruvate, sulfide, and ammonia. Despite this evidence, the biological context of *cdsH*’s role is not completely clear, especially in the infective cycle. Since inside epithelial cells both *cdsH* and its positive regulator, *ybaO*, are overexpressed, we hypothesized a possible role of *cdsH* in the intestinal phase of the infection. To test this hypothesis, we used an in vitro model of HT-29 cell infection, adding extra cysteine to the culture medium during the infective process. We observed that, at 6 h post-invasion, the wild type *S.* Typhimurium proliferated 30% more than the Δ*cdsH* strain in the presence of extra cysteine. This result shows that *cdsH* contributes to the bacterial replication in the intracellular environment in increased concentrations of extracellular cysteine, strongly suggesting that *cdsH* participates by increasing the bacterial fitness in the intestinal phase of the *S.* Typhimurium infection.

## 1. Introduction

Salmonellosis corresponds to one of the most common food diseases, affecting an estimated ten million people annually, with more than 100.000 deaths worldwide. The disease symptoms are noticeable between 6 and 72 h after consuming *Salmonella*, with a duration of 2–7 days [1].

*Salmonella enterica* serovar Typhimurium (*S.* Typhimurium) is one of the most relevant etiologic agents of salmonellosis. The infection begins with the consumption of contaminated food or water, where bacteria reach the distal ileum and induce their internalization into epithelial cells and M cells, among others, in the so-called intestinal phase of the infection [2]. *S.* Typhimurium produces self-limited gastroenteritis in healthy humans at this phase, whereas, in immunosuppressed individuals, children, and older adults, it can cause bacteriemia, or even a systemic infection in certain particular cases [3].

The small intestine epithelium’s primary function is to absorb nutrients from the lumen, since 80% of the total cells in this epithelium correspond to absorptive cells known as enterocytes [4]. Carbohydrates, fatty acids and proteins are biomolecules that can be uptaken by these cells. For their part, proteins are broken down into amino acids, capable of entering by active or secondary active transport, either as monomers or small peptides. The latter can be hydrolyzed inside the epithelial cells to become monomers [5,6].

Cysteine is a semi-essential amino acid for humans, belonging to the group of neutral polar amino acids. There are transporters for cysteine and cystine, an oxidized dimer form of cysteine, in the intestine epithelial cells. However, different systems are used for the absorption of these molecules from the intestinal lumen. For cysteine, the neutral transporter systems are used, such as the sodium-dependent ASC (alanine-serine-cysteine) system and symport or antiport mechanisms; for cystine, the transporters belong to the basic system, which transports cationic amino acids and cystine by means of an antiport mechanism independent of ions [7].

To our knowledge, the concentration of cysteine has not been reported for enterocytes. However, the intracellular cysteine levels reported for other cells were around 0.1–0.25 mM in eukaryotes [8]. The intracellular concentration of other amino acids in the jejunum’s epithelial cells is approximately 0.2–5 mM, with methionine having the lowest concentration [9]. On the other hand, the luminal concentration of amino acids (excluding cysteine) before food intake is 0.05–0.32 mM in the jejunum and between 0.6 and 0.86 µM in the ileum. In both cases, methionine has the lowest concentration [9]. Since methionine and cysteine have a similar presence in proteins, i.e., 2.3 and 1.9%, respectively [10], it is possible to infer that the increase in the concentration of these amino acids after consuming a diet rich in proteins should be similar in the intestinal lumen. A protein-rich diet increases methionine concentration from 0.05 to 0.6 mM in the jejunum and from 0.06 to 0.29 mM in the ileum [9], concentrations that plausibly are similar for cysteine.

In 2008, the gene encoding the inducible cysteine desulfhydrase enzyme, CdsH, was described, which produces pyruvate, ammonia and sulfide from cysteine degradation [11]. Under anaerobic conditions, a YbaO-dependent increase in *cdsH* expression has been shown in response to high cysteine concentrations (>0.2 mM). YbaO corresponds to a positive transcriptional regulator belonging to the Lrp family [11]. The range of cysteine concentrations in which CdsH would function in *Salmonella* (0.1–0.2 mM) suggests that this amino acid’s physiological concentration should be within these same limits, similar to what was described for *Escherichia coli* [12]. Interestingly, this cysteine concentration range allows bacteria to synthesize glutathione, a tripeptide that participates in defense against oxidative stress [13]. Besides, glutathione also generates hydrogen sulfide, which decreases the oxidative stress produced by certain antibiotics [14]. The synthesis of these defense compounds is explained by the fact that, at higher concentrations than physiological, cysteine is toxic in various microorganisms [12,15,16,17]. In *Salmonella*, excess cysteine induces a temporary toxic effect, causing a decreased growth in minimal media [18]. Many mechanisms explain the toxic effect of higher levels of cysteine. In both *E. coli* and *Salmonella*, the addition of cysteine has been shown to promote the Fenton reaction, with the consequent increase in the hydroxyl radical (·OH) in the presence of hydrogen peroxide, causing breaks in the DNA strands [12,17]. Another toxicity mechanism is related to the hindrance of obtaining or synthesizing branched-chain amino acids (BCAAs), such as valine, leucine, and isoleucine. In *E. coli*, cysteine has been shown to inhibit enzymes related to BCAA biosynthesis [19,20,21,22,23]. This mechanism is similar to that observed in some *Streptococcus mutans* strains, where the inhibitory effect on the bacterial growth under higher concentrations of cysteine is reversed by the addition of BCAA to the culture medium [16].

At present, the role of *csdH* in *S.* Typhumiurium’s infective cycle is not clear. A transcriptomic study of *S.* Typhimurium inside epithelial cells found that both *cdsH* and *ybaO* were overexpressed at different times post-infection. This evidence suggests that both genes play a role at this stage of the infection [24]. Due to the induction of *cdsH* expression inside epithelial cells and considering that this gene is overexpressed under high-cysteine concentrations, we wondered whether *cdsH* contributes to the *S*. Typhimurium replication inside epithelial cell lines in the presence of exogenous cysteine in vitro. We found that, at 6 h post-invasion, the wild type *S.* Typhimurium strain proliferates 30% more when extra cysteine is added to the medium, compared with the Δ*cdsH* strain in HT-29 cells. The contribution of *cdsH* to the replication of *S.* Typhimurium inside epithelial cells in response to cysteine is discussed.

## 2. Materials and Methods

Bacterial growth. Bacteria were grown at 37 °C in Luria–Bertani medium (tryptone, 10 g/L; yeast extract, 5 g/L; NaCl, 5 g/L) or glucose-supplemented M9 minimal medium (M9-glu) (Na_2_HPO_4_ × 7H_2_O, 12.8 g/L; KH_2_PO_4_, 3 g/L; NaCl, 0.5 g/L; NH_4_Cl, 1 g/L; MgSO_4_, 0.24 g/L; CaCl_2_, 0.011 g/L; glucose, 4 g/L, Merck^®^ KGaA, Darmstadt, Germany), and for solid medium agar-agar was added (15 g/L, Becton Dickinson^®^, Franklin Lakes, NJ, USA). When needed, antibiotics (Sigma-Aldrich^®^, Merck^®^ KGaA, Darmstadt, Germany) were added at the following concentrations: Ampicillin (Amp) 100 μg/mL, chloramphenicol (Cam) 20 μg/mL or kanamycin (Kan) 50 μg/mL.

Cell line culture. The HT-29 cell line was cultured in Dulbecco’s Modified Eagle Medium (DMEM) (Gibco^®^, ThermoFischer, Pittsburgh, PA, USA) supplemented with fetal bovine serum (Gibco^®^, ThermoFischer, Pittsburgh, PA, USA) (10%). Culture conditions were maintained at 37 °C in a humified atmosphere containing 5% CO_2_.

The construction of mutants and complemented strains of *S.* Typhimurium LT2. *S.* Typhimurium Δ*cdsH*::FRT or Δ*ybaO*::FRT were constructed as described by Datsenko and Wanner (2000) [25]. *S.* Typhimurium *cdsH*-3×FLAG and *ybaO*-3×FLAG mutants were constructed as described by Uzzau et al. (2001) [26]. The respective primers and strains are listed in Table 1 and Table 2, respectively. For complemented strains, primers were designed to amplify the promoter and coding regions of *cdsH* or *ybaO* (Table 1). Amplicons were cloned into the pBAD-TOPO (Invitrogen^®^, ThermoFischer, Pittsburgh, USA) vector following the manufacturer’s instructions, and then electrotransformed into *E. coli* DH5α. Then, the insert was released (EcoRI, New England Biolabs^®^, Ipswich, MA, USA) and subcloned into pACYCDuet (pACYC, Novagen^®^, Merck^®^ KGaA, Darmstadt, Germany, Cam^R^, P15A *ori*, intermediate/medium copy number) or pMCL210 (Cam^R^, P15A *ori*, intermediate/medium copy number). The resulting plasmids (pACYC::*cdsH* or pMCL210::*ybaO*) were used to complement the respective deletion (Table 2).

Growth curves. Growth curves were performed in LB and M9-glucose media with or without cysteine supplementation (0.2 mM). Bacteria were grown aerobically until the stationary phase, and optical density at 600 nm (OD_600_) was measured in a microtiter plate reader (Guangzhou Jet Bio-Filtration Co^®^, Guangzhou, China) every hour.

Cysteine minimum inhibitory concentration (MIC). An overnight culture of each strain was used to generate a new culture with approximately 1 × 10^4^ CFU/mL (the equivalent of a 0.5 McFarland standard). Bacterial cultures were disposed in 96-well microtiter plates and treated with decreasing concentrations of freshly prepared cysteine solutions. Microtiter plates were incubated in a Thermo shaker incubator (HILAB^®^ MSC-100, Curitiba, Brazil) at 37 °C with shaking for 16 h, and OD_600_ was measured in a microtiter plate reader. The MIC was defined as the lowest concentration that completely inhibited visible growth compared with the cysteine-free control. In other words, a lower MIC is interpreted as an increased susceptibility.

Cysteine toxicity assay (Kirby–Bauer Assay). To qualitatively assess the toxic effect of cysteine under aerobic conditions, both LB and M9-glucose plates were used. Overnight cultures were used to generate a 3 mL bacterial suspension in M9-glucose with agar-agar (0.7%) with approximately 10^6^ CFU. This bacterial suspension was added over an M9-glucose plate (agar-agar 1.5%) to generate a bacterial suspension layer. A filter-paper disc was then placed in the center of each bacterial layer before being loaded with 15 µL of a fresh solution of cysteine (0.5 M). The inhibition zone diameter was measured after 16 h incubation at 37 °C.

RNA isolation and mRNA detection. Total RNA extraction and RT–qPCR analyses were performed as previously described by Álvarez et al. (2015) [14]. Cultures of *S.* Typhimurium LT2 wild type and ∆*ybaO* mutant strain were grown in M9-glucose broth and incubated with shaking at 37 °C until OD_600_ = ~0.4. When necessary, bacterial cultures were exposed to 0.2 mM cysteine for 20 min. RNA was obtained by the acid–phenol method. Briefly, RNA was precipitated with isopropanol for 10 min at room temperature, washed with ice-cold 70% *v/v* ethanol and resuspended in DEPC-treated water before treating with 2U DNase I (Promega) to remove any DNA trace. The purity of the extracted RNA was determined by spectrometry (Absorbance 260/280). cDNA synthesis was carried out at 37 °C for 1 h in 25 µL of a mixture that contained 2.5 pmol of the specific reverse primers, 1 µg of template RNA, 0.2 mM dNTPs, 1 mL of nuclease-free water, 4 µL of M-MLV Reverse Transcriptase 5× Reaction Buffer (250 mM Tris-HCl pH 8.3, 375 mM KCl, 15 mM MgCl2, 10 mM 1,4-dithiothreitol (DTT), 40 U of RNasin) and 100 U of M-MLV reverse transcriptase (Promega^®^, Madison, WI, USA). The relative quantification of transcript levels was performed by RT–qPCR using the Brilliant II SYBR Green QPCR master reagent kit and the Mx3000P detection system (Stratagene^®^, San Diego, CA, USA). The 16s rRNA levels were used for normalization. The RT–qPCR mixture (20 µL) contained 50 ng of cDNA template, 120 nM each primer (cdsHRTF-cdsHRTR for *cdsH*, ybaORTF-ybaORTR for *ybaO*, and 16SF–16SR for 16s gene, see Table 1) and 1 µL of ROX reference dye (1:200). RT–qPCR was performed under the following conditions: 10 min at 95 °C followed by 40 cycles of 15 s at 95 °C, 15 s at 60 °C, and 25 s at 72 °C, followed by a melting cycle from 65 to 95 °C to check for amplification specificity. A previous standard quantification curve with serial dilutions of RT–qPCR products was constructed for each gene to calculate the amplification efficiency. These values were used to obtain the ratio between the gene of interest and the 16s rRNA gene expression. The experiment was performed for three biological and technical replicates. These data were analyzed with the MxPRO software (Agilent Technologies^®^, Santa Clara, CA, USA).

Western blot. Total protein extraction was performed as previously described by Nevermann et al. (2019) [27]. Briefly, cultures of *S.* Typhimurium LT2 wild type and ∆*ybaO* mutant strain were grown in M9-glucose broth and incubated with shaking at 37 °C until OD_600_ = ~0.4. When necessary, bacterial cultures were exposed to cysteine 200 µM for 1 h before centrifugation, and the pellet was resuspended in Tris-HCl 100 mM buffer. Total proteins were extracted by sonication on ice (30 s pulses, amplitude 70%) with sonicator Vibra-Cell. Protein quantification was performed with the classical Bradford method [28].

Western blot was performed as previously described by Jofre et al. (2014) [29]. Briefly, 100 µg of protein extracts of each sample were loaded by SDS-PAGE (12% acrylamide). Proteins were transferred to a nitrocellulose membrane (Bio-Rad) and blocked overnight at 4 °C with milk in TBS (1.4% NaCl, 0.6% glycine pH 7.4). The membrane was washed three times with TBS-Tween (NaCl 1.4%, glycine 0.6%, Tween 0.1%). The western blots were probed with a 1:5000 dilution of primary antibody anti-FLAG for 12 h at 4 °C. The membrane was rewashed with TBS-Tween and then probed with a mix of 1:10,000 dilution of secondary antibody anti-mouse-horseradish peroxidase (HRP) conjugate (Promega) and 1:25,000 dilution anti-mouse-HSP for 2 h with shaking at room temperature. Finally, the membrane was washed with TBS-Tween prior to being revealed by the BCIP/NTB (Invitrogen^®^) kit according to the manufacturer’s instructions. Hsp60 was used as a control load, as described [30]. The Western blot was repeated at least three independent times and analyzed by densitometry to quantify the relative amounts of protein by ImageJ Software.

Deoxycholate survival assay. A 25 mL OD_600_ ~0.5 culture of each strain growing on LB broth was washed three times with phosphate saline buffer (PBS) and finally resuspended in 5 mL of PBS. Then, 20 µL of each bacterial suspension was resuspended in a 96-well microplate with 180 µL of deoxycholate (Sigma-Aldrich^®^, Merck^®^ KGaA, Darmstadt, Germany) 0.5% or PBS (control) before incubating them for 2 h. Finally, each well was serially diluted in PBS and seeded in LB plates for CFU counting.

Bacterial viability in the HT-29 cell line with extra supplementation of cysteine. Cell viability was determined by neutral red uptake assay, as previously described by Repetto et al. (2008) [31]. Briefly, HT-29 cell cultures (DMEM) were washed with PBS and refilled with DMEM alone or supplemented with cysteine. This procedure was repeated at 1 h and 3 h of incubation. After 24 h of incubation, cells were washed with PBS, and a neutral red solution diluted in DMEM (Sigma-Aldrich^®^, Merck^®^ KGaA, Darmstadt, Germany) (60 µg/mL) was added before incubation at 37 °C for 2 h. Next, each well was washed twice with PBS, and a destaining solution (50% ethanol (Merck^®^ KGaA, Darmstadt, Germany) 49% deionized water, 1% glacial acetic acid (Merck^®^ KGaA, Darmstadt, Germany)) was used to remove the neutral red solution from cells. Finally, OD_540_ was measured for each well. A well was treated with deoxycholate 0.5% for 5 min before neutral red was added as a control.

Epithelial cell line replication assay. *S.* Typhimurium LT2 invasion and replication were determined using the gentamycin-protection assay described by Fuentes et al. (2008) with some modifications [32]. Briefly, the *S.* Typhimurium strains used to infect HT-29 monolayer were grown in microaerophilic conditions to an OD_600_ = 0.2 at 37 °C. Then, bacteria were washed three times with PBS and finally resuspended in DMEM with or without cysteine (0.2 mM). This suspension was immediately used for HT-29 monolayer infection in 96-well plates using a multiplicity of infection of 100:1. One hour after infection, cells were washed three times with PBS and a DMEM solution supplemented with gentamycin (200 µg/mL), with or without cysteine, was added to each well. The previous procedure was repeated three hours after infection but without gentamycin, and infected cells were incubated for 3, 6 or 24 h post-infection (p.i.). To assess bacterial invasion and replication, the CFU were determined in each time and condition. Bacteria were recovered from HT-29 cells at 3 (invasion), 6 and 24 h (replication) p.i. using a deoxycholate solution (0.5% in PBS) and mechanical disruption. The bacterial suspension was seeded in LB plates, and CFU were counted after 16 h of incubation at 37 °C.

Statistical analysis. All analyses were performed using IBM SPSS and GraphPad Prism 8 software. ANOVA with Tuckey’s test as post hoc and Mann–Whitney tests were performed in the cysteine Kirby–Bauer and mRNA expression experiments, respectively. Two-way ANOVA with post hoc Tukey’s multiple comparisons test was performed for the replication experiment analysis.

## 3. Results

### 3.1. The cdsH and ybaO Genes Participate in the Response of S. Typhimurium against High Concentrations of Cysteine in Minimal Medium

**Figure 1:**
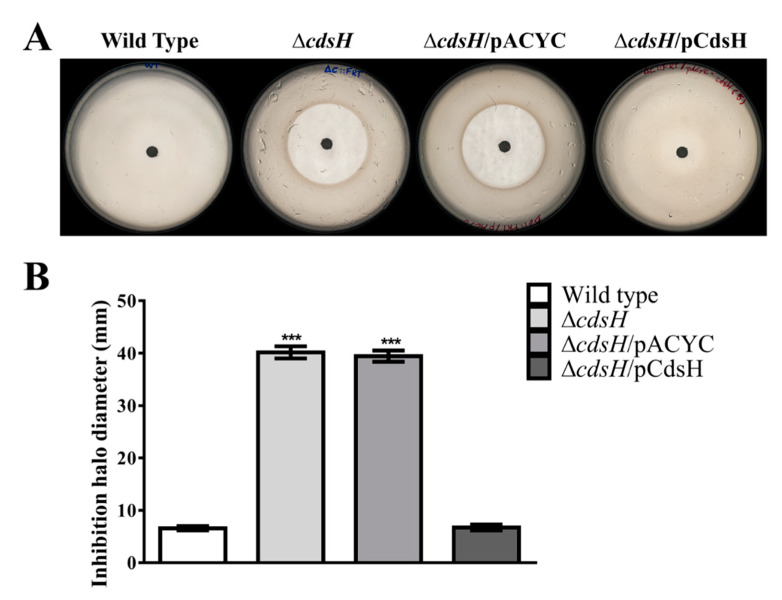
Inhibitory effect of cysteine in minimal medium (M9-glucose) under aerobic conditions. Overnight cultures in M9-glucose of *S*. Typhimurium strains were used to generate an agar-agar (0.7%) layer with approximately 10^6^ CFU. A filter-paper disc was placed over the layer and loaded with 15 µL of 0.5 M cysteine solution. (**A**) Inhibition haloes generated by cysteine on wild type, Δ*cdsH* mutant, Δ*cdsH*/pACYC (empty vector), and Δ*cdsH*/pCdsH (complemented strain). (**B**) Graphic representation of inhibition haloes generated by cysteine in A). ANOVA test (post hoc: Tuckey’s test) was performed (error bars represent standard error of the mean, *n* = 7; ***, *p* < 0.001 in comparison with the wild type strain).

Since CdsH responds to an excess of cysteine and YbaO controls the expression of CdsH under anaerobic conditions [11], we wondered whether *S.* Typhimurium Δ*cdsH* exhibited increased susceptibility to exogenous cysteine under aerobic growth. For this aim, a Kirby–Bauer test was carried out in M9-glucose minimal medium, a culture medium that does include cysteine. A cysteine solution of 0.5 M was added to a filter-paper disc placed on the M9-glucose minimal medium plates, previously seeded with the tested bacteria, before incubation for 16 h at 37 °C to obtain growth inhibition haloes. Growth inhibition haloes of approximately 40 mm diameter were observed for the mutant strains, while the wild type exhibited no growth inhibition. The mutant phenotype was fully complemented by the corresponding plasmid (Figure 1). This result argues for the role of *cdsH* in the resistance to high cysteine concentrations.

In order to set the sublethal concentration of cysteine for the following experiments, the MIC of cysteine was determined for the *S.* Typhimurium wild type strain and the mutant derivatives (Table 3). Compared to the wild type strain, mutant strains (and mutants harboring the empty vector) showed a 16-fold lower MIC. On the other hand, the respective complemented mutants showed MICs even higher than those presented by the WT, plausibly due to the increased gene dosage provided by the respective cloning vector (Table 3). All these results support the participation of both *cdsH* and *ybaO* in cysteine susceptibility under the tested conditions. Based on these results, we set the work concentration of cysteine to 0.2 mM, half of the MIC regarding *S.* Typhimurium Δ*csdH* and Δ*ybaO* (Table 3). This concentration is similar to the higher concentration inferred for cysteine in the ileum (as extrapolated from the methionine concentration, see above) [9]. As a control, and to determine any additional detrimental effects of cysteine, we analyzed the growth curve of *S.* Typhimurium wild type and mutant derivatives, either in M9-glucose supplemented with 0.2 mM cysteine or in LB (rich medium) supplemented with 0.2 mM cysteine (37 °C with shaking). As shown in Figure S1, we found no differences in the growth curves of the wild types and mutants in LB, despite the presence of cysteine. In the case of M9, we observed a slightly extended lag phase, although no differences in the growth rate (μ) were observed. No differences were observed when bacteria were cultured in the absence of supplemented cysteine (Figure S2). These results show that the addition of 0.2 mM of exogenous cysteine exerts no detrimental effects in the *S.* Typhimurium Δ*cdsH* or Δ*ybaO* in a rich medium (LB). By contrast, in a poorer environment (i.e., minimal medium M9), the lack of either *cdsH* or *ybaO* seems to slightly delay the adaptation of strains, although no significant effects in the growth rate were observed under the tested conditions (Table S1).

### 3.2. The Expression of the cdsH Gene is Induced in the Presence of Cysteine

**Figure 2:**
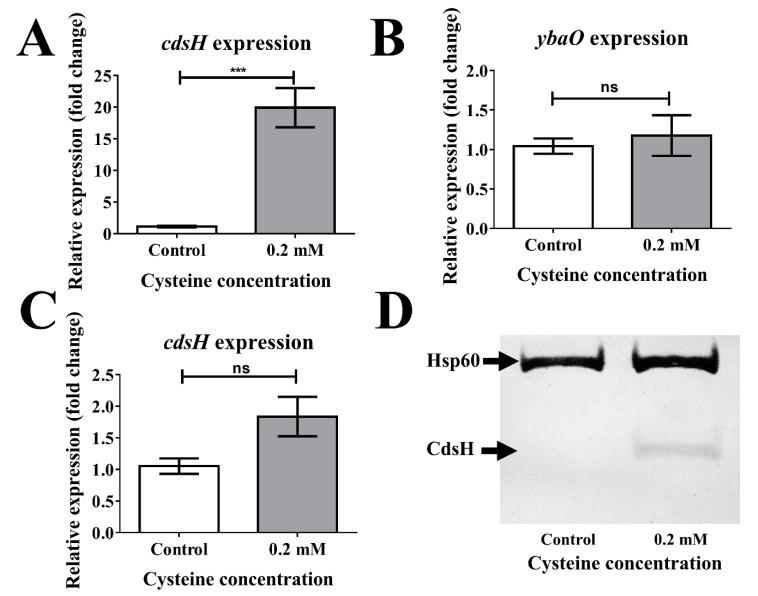
Aerobic *cdsH* and *ybaO* expression in response to cysteine. *S.* Typhimurium strains were grown in M9-glucose to OD_600_ 0.4–0.5 prior to supplementing with cysteine (0.2 mM, final concentration) or water (control). After 20 min incubation, bacteria were harvested, and an RT–PCR assay was performed as described in Materials and Methods (**A**–**C**). (**A**) *cdsH* expression in the WT context. (**B**) *ybaO* expression in the WT context. (**C**) *cdsH* expression in the Δ*ybaO* context. A-C, Mann–Whitney test (error bars represent standard error of the mean, *n* = 4, ns, no significative; ***, *p* < 0.001). For the Western blot assay (**D**), the *S.* Typhimurium *cdsH*-3×FLAG was cultured in M9-glucose to OD_600_ 0.4–0.5 prior to supplementing with cysteine (0.2 mM, final concentration) or water (control). Hsp60 was used as a control load, as described [30]. After 1 min incubation, bacteria were harvested, and the Westen blot assay was performed as described in Materials and Methods (a representative result of *n* = 3).

Since our results show that *cdsH* and *ybaO* participate in the adaptation to the presence of exogenous cysteine, we explored whether this amino acid induces the expression of these genes. For this purpose, RT–qPCR and Western blot assays were carried out from cultures in M9-glucose medium alone (control) or M9-glucose supplemented with 0.2 mM cysteine. As shown in Figure 2, the addition of cysteine induced the expression of *cdsH* only when the *ybaO* gene was present (Figure 2), corroborating the previous work of Oguri et al. (2012) [11]. By contrast, in a Δ*ybaO* context, this induction was not observed (Figure 2C). Regarding the expression of *ybaO*, we found that, under the tested condition, the addition of cysteine produced no effect compared with the M9-glucose alone (Figure 2B), which can be explained by the negative self-regulation of the LRP regulator family [33].

The *S*. Typhimurium *ybaO-*3×FLAG strain was also used to assess the *ybaO* expression at the protein level. Nevertheless, the Western blot assay could not detect this protein, even in the presence of cysteine (data not shown), strongly suggesting that the amount of YbaO is too low to be detected by the performed procedure. All these results agree with that previously described by Oguri et al. (2012) [11], who assessed the *cdsH* expression with a nutrient-deficient medium but under anaerobic conditions.

Altogether, these results show that *cdsH* increased its expression in the presence of cysteine in a YbaO-dependent manner, even under aerobic conditions. Since *ybaO* showed no increase in its transcription or translation in the presence of cysteine, unlike what was observed for *cdsH*, we decided to continue assessing only the participation of *cdsH*.

### 3.3. Cysteine Promotes Replication of S. Typhimurium inside Epithelial Cells in a cdsH-Dependent Manner

According to the transcriptomic analysis carried out by Hautefort et al. (2008), *cdsH* is overexpressed when *Salmonella* is inside epithelial cells. Since this gene is induced in response to the presence of harmful cysteine concentrations, we wondered whether the increased *cdsH* expression is related to a bacterial defense response inside epithelial cells. To this end, both the invasion and the replication of *S.* Typhimurium strains were assessed in the HT-29 cell line, comparing the standard DMEM medium with DMEM supplemented with extra cysteine (0.2 mM).

**Figure 3:**
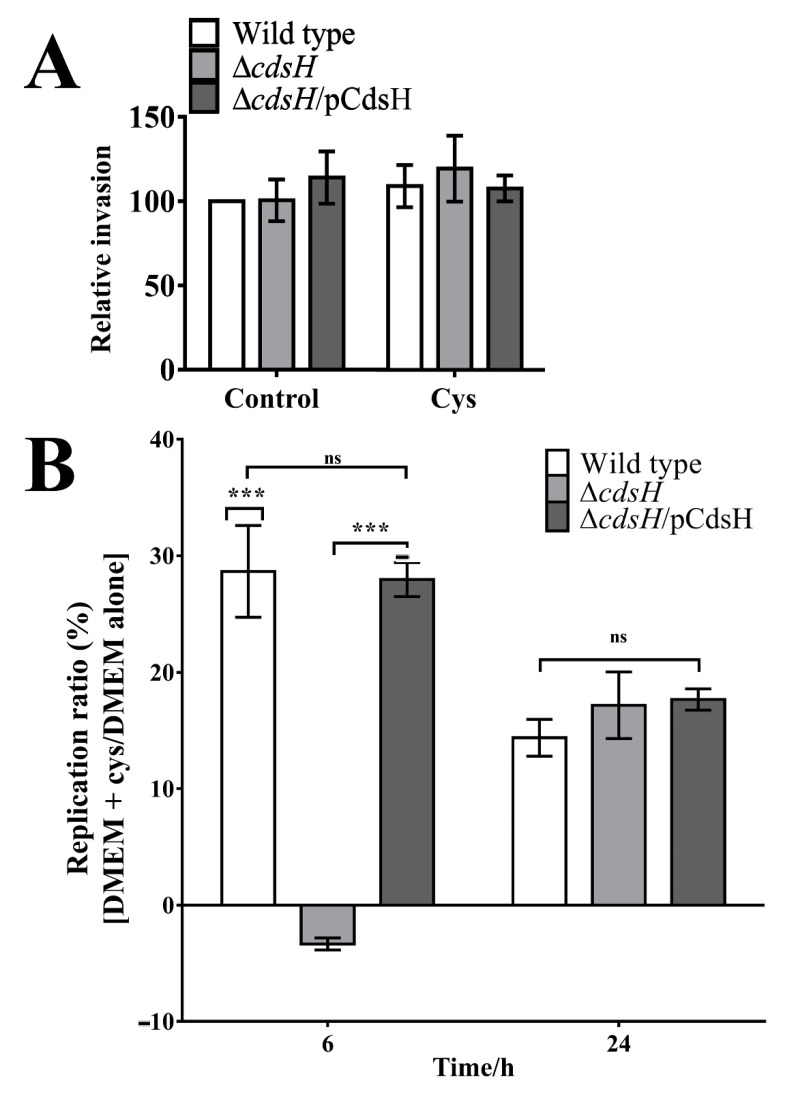
*S.* Typhimurium replication in HT-29 cell line with and without cysteine. HT-29 cell line cultures in 96-well plates were infected with wild type, Δ*cdsH*, or Δ*cdsH*/pCdsH strains in DMEM medium alone or supplemented with 0.2 mM cysteine. (**A)** Three hours post-infection (relative invasion related to the wild type strain); (**B**) Six and twenty-four hours post-infection (early and late replication). The figure shows the percentage replication ratio of DMEM + cys:DMEM alone. Bacteria were recovered from the intracellular environment by disrupting cells with 0.5% sodium deoxycholate prior to plating on LB agar to count CFU. Two-way ANOVA (post hoc: Tukey) was performed for analysis (error bars represent standard error of the mean, *n* = 5; ns, no significance; ***, *p* < 0.001).

Since HT-29 cells are typically cultured in standard DMEM medium, we first assessed whether the supplementation with additional cysteine (0.2 mM) affects cell viability. To that aim, we performed a neutral red uptake [31]. As shown in Figure S3A, extra cysteine in the DMEM medium produced no effects on the HT-29′s viability. Similarly, we found that the DMEM supplemented with additional cysteine exerted no detrimental effects on the growth of *S.* Typhimurium and derivatives, even after an incubation of 3, 6, 12, or 24 h (Figure S3B), in agreement with what was found with LB supplemented with cysteine (Figure S1). Having ruled out possible artifactual results, we tested the invasion and replication of *S.* Typhimurium and mutant derivatives in either DMEM or DMEM supplemented with cysteine by the gentamicin protection assays with HT-29. As shown in Figure 3A, we found indistinguishable invasions between the *S.* Typhimurium wild type and Δ*cdsH*, with or without additional cysteine. By contrast, the addition of extra cysteine to the DMEM medium slightly decreased the replication of Δ*cdsH* bacteria, while it increased the wild type strain replication up to 30% at 6 h, but not at 24 h, where the number of intracellular bacteria similarly increased for all the strains, even with the addition of extra cysteine cultures (Figure 3B). As shown in Table 4, the generational time of intracellular bacteria was longer in the Δ*cdsH* strain only when extra cysteine was added, supporting our results.

All these results together argue for the participation of *cdsH* in *S*. Typhimurium replication within epithelial cells in high cysteine concentrations. Thus, *cdsH* contributes to improving the bacterial fitness inside epithelial cells.

## 4. Discussion

*Salmonella* is an enteric pathogen that produces a wide variety of diseases, from self-limited gastroenteritis to systemic infections and death. During the infective process, before the internalization into epithelial cells, *Salmonella* overcomes different stressful conditions such as acid pH, bile salts, high osmolarity, and low oxygen tension [2]. Inside epithelial cells, *Salmonella* responds to the intracellular environment to achieve a successful replication, which is an essential process for the bacteria’s virulence and develops a successful infection [34]. Since intestinal epithelial cells are continually absorbing nutrients from the lumen, the intracellular amino acid concentration increases [9]. Under these conditions, intracellular bacteria could use these high amino acid concentrations for their own purpose. In this context, *S*. Typhimurium has a cysteine desulfhydrase enzyme, CdsH, that responds to high cysteine concentrations to avoid the toxicity of this amino acid. As *cdsH* increases its expression inside epithelial cells [24], *S.* Typhimurium may use this enzyme to prevent the toxic effect, or use cysteine to increase its fitness.

### 4.1. Cysteine Susceptibility Occurs only in Minimal Medium

It has been shown that cysteine affects the growth of multiple bacteria in minimal media, and *S*. Typhimurium is not an exception. *S.* Typhimurium is also susceptible to the toxic effect of excess cysteine in minimal media, although this is not the case in a rich medium (Figure S1 and Figure S3). The cysteine susceptibility increases when the CdsH enzyme cannot be synthesized in the Δ*cdsH* mutant, or as a result of the absence of its positive regulator YbaO in the Δ*ybaO* context (Figure 2 and Table 3). When *S*. Typhimurium grows in minimal media, the expression of the genes involved in the transport of molecules is increased [35]. Accordingly, under sulfur-limited conditions, bacteria respond by synthesizing CysB-regulated sulfide transporters [36]. Thus, cysteine can enter bacteria in multiple ways, including derivatives such as cystine. For instance, in *E. coli*, extracellular cystine increases the intracellular cysteine concentration, exerting a toxic effect [12,17]. Therefore, the addition of both exogenous cysteine and cystine to the culture medium increases the concentration of intracellular cysteine when *Salmonella* is in a nutrient-deficient environment, and would cause the toxic effect produced by this amino acid. This could be explained by the differences in the global expression patterns when bacteria grow in nutrient-rich media versus nutrient-deficient media. For example, in *E. coli*, the number of transport genes overexpressed in minimal media is higher than the number of transport genes overexpressed in a rich medium when the expression is compared between both media [35]. Considering that cysteine enters the cell by specific transporters, the overexpression of these transporters could explain why, in minimal media, cysteine is toxic in lower concentration than in rich media.

Under aerobic conditions, we observed similar results to those previously reported in anaerobic conditions by Oguri et al. (2012) [11], i.e., *S*. Typhimurium is susceptible to excess cysteine only in minimal medium (Figure S2), and *cdsH* is overexpressed in response to the addition of exogenous cysteine to the medium in a YbaO-dependent manner, this being an Lrp family regulator that does not change its expression in response to cysteine.

### 4.2. CdsH Contributes to the Replication of S. Typhimurium in the HT-29 Line, against the Addition of Exogenous Cysteine

As mentioned before, during the invasion of the intestine, *Salmonella* is exposed to different conditions [37]. These conditions produce changes in bacterial genetic profile, promoting the invasion and subsequent replication inside epithelial cells [38,39]. In both processes, the effector and regulatory genes from *Salmonella* pathogenicity island I and II are important, since mutation on these genes produces non-invasive phenotypes [40]. In the replication assay, at 3 h post-infection, no differences between wild type and Δ*cdsH* strains were observed in both DMEM and DMEM + cysteine media (Figure 3A). Since the replication of *Salmonella* inside epithelial cells begins between 3 and 4 h after the invasion [41,42], this process was not affected by *cdsH* deletion in both conditions; therefore, *cdsH* does not contribute to the invasion of epithelial cells. This result is in accordance with the function of CdsH, which participates in the elimination of cysteine, and its expression is regulated in that context, unlike the expression of genes such as *invA* and *sipA*, among others, whose expression is directly related to the *Salmonella*-invasion of epithelial cells. Therefore, according to our results, CdsH does not participate in the invasion process.

Even though replication within epithelial cells mainly depends on the genes present in SPI-1 and SPI-2 [43,44], genes involved in other processes can also contribute to bacterial replication within this cell type. An example of this was reported in *S*. Typhi, where the mutation in genes related to anaerobic respiration causes lower bacterial replication inside epithelial cells, possibly because these genes would allow bacteria to perform more energy-efficient metabolic processes [45]. Other genes could also be indirectly related to the replication process, such as *trxA* and *trxB*, components of the thioredoxin pathway (a protein that acts as an antioxidant), where a mutation in these genes slightly decreases replication inside of epithelial cells [46].

In other cell types, non-classical factors that contribute to *Salmonella* replication have also been seen. The Tsx porin participates in nucleoside uptake, where a mutation in this gene has been shown to decrease *Salmonella*’s replicative capacity inside a line of macrophages [47]. Likewise, the *S.* Typhimurium ∆*cdsH* strain’s replication does not respond to the addition of cysteine to the medium as the wild type strain at 6 h (Figure 3B). Since this gene is overexpressed in the presence of exogenous cysteine [11,48,49] and cystine [50], overexpression within epithelial cells [24] can be attributed to the entry of cysteine or cystine into bacteria. Although culture media do not have cysteine, they do have cystine. In this sense, the increased *cdsH* expression may be due to cystine uptake by bacteria. Related to this, YdjN, one of the primary transporters for cystine, was also found to have an increased expression within epithelial cells [24]. When comparing the replication of each strain in the supplemented versus the non-supplemented medium at different times, we found that the wild type strain exhibits an advantage in replication at 6 h p.i. when cysteine is added to the medium, increasing the replication. An explanation could be the pyruvate synthesis from cysteine by CdsH [11], which contributes to bacteria’s energy production. In addition, it has been shown that *Salmonella* can use CdsH to synthesize thiamine, in a different way from the conventional, which is transformed into thiamine pyrophosphate, a coenzyme required for enzymes of the central metabolism [51]. In fact, in *Listeria monocytogenes,* it has been shown that replication inside epithelial cells requires the uptake and biosynthesis of thiamine precursors [52]. Thus, this could represent an advantage of the wild type over the Δ*cdsH* strain. On the other hand, at 24 h p.i., the mutant strain was able to use cysteine, reaching a replication ratio comparable to that of the wild strain, thus suggesting the existence of another redundant system that can fulfill this function. In this sense, it is known that other enzymes such as CysK or CysM also have cysteine desulfhydrase activity, although these are not cysteine-inducible [11].

Cysteine can exert its toxic activity mainly by producing oxidative stress through the reduction of Fe(III) to Fe(II) and the subsequent stimulation of the Fenton reaction, as well as hydroxyl radical production [12]. In this context, CdsH has been described as the major cysteine-degrading and sulfide-producing enzyme [11]. The degradation of cysteine could be the more immediate response in order to prevent the cysteine-dependent ROS. Nevertheless, in the absence of CdsH and ROS’s eventual appearance, other mechanisms to circumvent the insult are plausibly triggered [53]. Besides, cysteine concentration might decrease over time due to its consumption in anabolic processes. In this context, the role of CdsH could be circumscribed to more early stages, providing a possible explanation of why the lack of CdsH does not affect bacterial replication at 24 h post-infection (Figure 3).

In addition to classic virulence factors, other factors related to various bacterial processes, such as molecule transport, protein synthesis, and metabolism under certain conditions, contribute to bacterial replication inside the host cells and thus favor the virulence. In this context, CdsH promotes bacterial replication, at least in an in vitro assay, when it is internalized in epithelial cells in a medium with extra cysteine, which could partially simulate the intestinal environment after consumption of a high-protein meal.

## Figures and Tables

**Table 1:** Primers used in this study.

Primers	Comment	Sequence (5′-3′)
cdsHwannerF	Generation of Δ*cdsH*	GGCACAAAATGATAAATGGATATTAATGATGAGTAGCAATGTAGGCTGGAGCTGCTTCGA
cdsHwannerR	Generation of Δ*cdsH*	ATTCAGTCTGGCTTTTTTTTGGTTTCTAGTCGCCGGTAAGATATGAATATCCTCCTTAG
ybaOwannerF	Generation of Δ*ybaO*	GAAAAATATTCTCTATGGAGTGGGTATGTTAGATAAAATTGTAGGCTGGAGCTGCTTCGA
ybaOwannerR	Generation of Δ*ybaO*	ATTTCCGGCAATACCGGGAGAATTATTCAATGGGCAGAGACATATGAATATCCTCCTTAG
cdsHextF	Test the generation of Δ*cdsH*	AATAAGCAAAGCAGCTTACGGTCAA
cdsHextR	Test the generation of Δ*cdsH*	CACACCTTTCGGTTATCGGACATTG
ybaOextF	Test the generation of Δ*ybaO*	ATCCATTTATCATTTTGTGCCAAGA
ybaOextR	Test the generation of Δ*ybaO*	GCCAAGATAGCGACGCCATT
cdsH3×FLAGF	Generation of *cdsH*-3×FLAG	GCCGTGGTCAGCGGCCATCGCAAAATTACTTACCGGCGACGACTACAAAGACCATGACGG GACTACAAAGACCATGACGG
cdsH3×FLAGR	Generation of *cdsH*-3×FLAG	ATTGGCTGGACAATCCGCATCTACCTTATTCCCCCGAATGCATATGAATATCCTCCTTA*G*
ybaO3×FLAGF	Generation of *ybaO*-3×FLAG	GATGGAACAGATTAAGTACACCACCTCTCTGCCCATTGAAGACTACAAAGACCATGACGG
ybaO3×FLAGR	Generation of *ybaO*-3×FLAG	CGACGCCATTCCCGGCGAAAGTACCAGCTTAATTGAGCAACATATGAATATCCTCCTTAG
cdsHpMCL210F	Cloning of *cdsH*	GAATTCTCAGATTAACGGCATCCGCC
cdsHpMCL210R	Cloning of *cdsH*	AAGCTTCTAGTCGCCGGTAAGTAATTTTGC
ybaOpMCL210F	Cloning of *ybaO*	GAATTCAGGGGCAAACGAATAAGATGCG
ybaOpMCL210R	Cloning of *ybaO*	AAGCTTTTATTCAATGGGCAGAGAGGTGG
cdsHRTF	RT–PCR	CGGCTGGCGCATGAACTGAA
cdsHRTR	RT–PCR	TTCCGGCGCTCATGACGATAA
ybaORTF	RT–PCR	TGGAAGATGACGGTATCCTGCTTGG
ybaORTR	RT–PCR	CCAAGCACTTCCGGCATCTCAGAG
16SF	RT–PCR	GTAGAATTCCAGGTGTAGCG
16SR	RT–PCR	TTATCACTGGCAGTCTCCTT

**Table 2:** Strains used in this study.

Genotype	Antibiotic Resistance	Source
*S.* Typhimurium LT2	-	ATCC^®^700720
*S.* Typhimurium LT2 Δ*cdsH*::FRT *	-	This study
*S.* Typhimurium LT2 Δ*cdsH*::FRT/pACYC	Cam^R^	This study
*S.* Typhimurium LT2 Δ*cdsH*::FRT/pACYC::*cdsH* **	Cam^R^	This study
*S.* Typhimurium LT2 Δ*ybaO*::FRT ***	-	This study
*S.* Typhimurium LT2 Δ*ybaO*::FRT/pMCL210	Cam^R^	This study
*S.* Typhimurium LT2 Δ*ybaO*::FRT/pMCL210::*ybaO* ****	Cam^R^	This study
*S.* Typhimurium LT2 *cdsH*-3×FLAG	Kan^R^	This study
*S.* Typhimurium LT2 *ybaO*-3×FLAG	Kan^R^	This study

* This strain is also called Δ*cdsH.* ** This plasmid is also called pCdsH. *** This strain is also called Δ*ybaO.* **** This plasmid is called pYbaO.

**Table 3:** MIC for cysteine in M9-glucose medium.

Strain	MIC (mM)
*S.* Typhimurium LT2	6.4
*S.* Typhimurium LT2 Δ*cdsH*	0.4
*S.* Typhimurium LT2 Δ*cdsH*/pACYC	0.4
*S.* Typhimurium LT2 Δ*cdsH*/pCdsH	>12.8
*S.* Typhimurium LT2 Δ*ybaO*	0.4
*S.* Typhimurium LT2 Δ*ybaO*/pMCL210	0.4
*S.* Typhimurium LT2 Δ*ybaO*/pYbaO	12.8

**Table 4:** Generation time at 24 h of the strains within the HT-29 line under normal conditions and with the addition of exogenous cysteine.

Strain	Generation Time without Cysteine (h)	Generation Time with 0.2 mM Cysteine (h)
*S.* Typhimurium LT2	12.64	10.01
*S.* Typhimurium LT2 Δ*cdsH*	16.90	28.46
*S.* Typhimurium LT2 Δ*cdsH/*pCdsH	16.84	16.56

## Supplementary Materials

The following are available online at https://www.mdpi.com/2076-2607/8/12/2019/s1, Figure S1: Growth curve in LB and M9-glucose; Figure S2. Growth curve with cysteine in LB and M9-glucose; Figure S3. Controls for cysteine supplemented replication assay; Table S1. Growth rate μ (h-1) in different culture media.
